# STRN3 promotes tumour growth in hepatocellular carcinoma by inhibiting the hippo pathway

**DOI:** 10.1111/jcmm.18147

**Published:** 2024-03-01

**Authors:** Jie Zhu, Wenjia Tang, Peiqi Fang, Chong Wang, Meixiu Gu, Wenjing Yang, Baishen Pan, Beili Wang, Wei Guo

**Affiliations:** ^1^ Department of Laboratory Medicine, Zhongshan Hospital Fudan University Shanghai China; ^2^ Department of Laboratory Medicine, Shanghai Geriatric Medical Center, Zhongshan Hospital Fudan University Shanghai China; ^3^ Department of Laboratory Medicine, Xiamen Branch, Zhongshan Hospital Fudan University Xiamen China; ^4^ Department of Laboratory Medicine, Wusong Branch, Zhongshan Hospital Fudan University Shanghai China

**Keywords:** hepatocellular carcinoma, hippo pathway, STRN3, TCGA database, YAP

## Abstract

HCC is a globally high‐incidence malignant tumour, and its pathogenesis is still unclear. Recently, STRN3 has been found to be elevated in various tumours, but its expression and biological functions in HCC have not been studied. In the study, clinical correlation analysis was performed on 371 liver cancer patients from TCGA database and liver cancer tissues and normal tissues from the GEO database. qRT‐PCR and western blotting were used to detect relevant proteins in cells, and CCK8 and colony formation experiments were performed to analyse cell proliferation ability. Transwell and wound healing experiments were performed to detect cell invasion ability, and flow cytometry was used to detect cell apoptosis. Single‐cell sequencing data and multiple immunofluorescence were analysed for the expression abundance and distribution of certain proteins. Immunohistochemistry was used to assess the expression of STRN3 in patients' tumour and adjacent non‐cancerous tissues. The results indicated STRN3 was highly expressed in liver tumour tissues and was closely associated with poor prognosis. Knockdown of STRN3 could significantly inhibit cell proliferation and migration ability. At the same time, we found that STRN3 could inhibit the Hippo pathway and promote the entry of YAP protein into the nucleus. Our study first found that STRN3 could promote tumour growth by inhibiting the Hippo pathway. The study of STRN3 can promote the understanding and treatment of the occurrence and development of HCC.

## 1 INTRODUCTION

Hepatocellular carcinoma (HCC) is a highly prevalent and lethal form of cancer that is responsible for the most cancer‐related deaths globally,^1^ and is considered as one of the lowest 5‐year relative survival rate for all cancers.^2^ Despite improved and novel developed treatment, HCC remains a challenging disease to manage due to its insidious development, high rate of recurrence and distant metastasis,^3^ which lead to a great proportion of patients are already in advanced stage at the time of diagnosis. In recent years, advances in genomics and transcriptomics have contributed significantly to the understanding of the molecular alterations and pathways involved in HCC.^4^ Several critical tumour driver genes have been proposed and identified as druggable targets, such as VEGFR (vascular endothelial growth factor receptors), FGFR (fibroblast growth factor receptors) and TGFA (transforming growth factor‐alpha).^5^ However, the underlying mechanisms of pathogenesis are not yet fully understood, making it challenging to develop effective treatment strategies and therapeutic targets.

Striatin 3 (STRN3) is a member of the striatin family proteins (STRN, STRN3 and STRN4), characterizing as B^′′′^ regulatory subunit of serine/threonine‐protein phosphatase 2A (PP2A), a critical component of multi‐component complexes known as striatin interacting phosphatase and kinase (STRIPAK) involved in various cellular processes.^6^, ^7^ Previous research has shown that STRN3 functions as a scaffold protein and is up‐regulated in several types of cancer, including gastric and breast cancer.^8^, ^9^ Studies have also demonstrated that STRN3 regulates the activation of the YAP (Yes‐associated protein) and hippo pathway, as well as being a vital upstream regulator of MAP4K4 to enhance cancer cell invasion and growth.^8^, ^10^ However, the specific function of STRN3 in HCC remains unknown.

Herein, in this study, we aimed to evaluate the expression features of STRN3 in HCC and explore its prognostic significance and association with clinical characteristics. Furthermore, the study aims to provide a thorough investigation of the biological function of STRN3 in HCC development. Through this research, a better understanding of STRN3's role in HCC can be achieved, which may lead to the development of novel therapeutic strategies for the treatment of this deadly disease.

## 2 MATERIALS AND METHODS

### 2.1 Patient enrolment and follow‐up

Twenty‐four HCC patients who underwent curative resection at Zhongshan Hospital from August 2021 to December 2021 were enrolled, and paratumoral and tumoral tissues were collected. All patients were diagnosed with HCC according to Guidelines for the Diagnosis and Treatment of Hepatocellular Carcinoma (2019 Edition). The observation period started from the initiation of surgical treatment until the patient's death or the last follow‐up visit. Each patient underwent serum testing, including alpha‐fetoprotein (AFP), alanine aminotransferase (ALT) and aspartate aminotransferase (AST), and also received imaging examinations, including CT or MRI scans 4–6 weeks after surgery, followed by additional scans every 2–3 months. This research has been approved by the research ethics committee of Zhongshan Hospital, Fudan University. The written informed consent was obtained from every patient in this study.

### 2.2 DATA acquisition

The clinical information, RNA‐sequence and follow‐up data of 371 HCC patients were obtained from The Cancer Genome Atlas (TCGA) (https://portal.gdc.cancer.gov). Genome‐wide gene expression profiles of 39 tumour tissues with 15 para‐tumour tissues (GSE65372) and 70 tumour tissues with 70 para‐tumour tissues (GSE144269) were downloaded from NCBI Geo DataSets(https://www.ncbi.nlm.nih.gov/gds/). Single‐cell RNA sequencing profile of HCC tissue (GSE210679) was obtained from NCBI Geo DataSets (https://www.ncbi.nlm.nih.gov/gds/). The Immunohistochemistry (IHC) samples stained with STRN3 in normal and cancerous human liver tissues were obtained separately from the Human Protein Atlas (HPA) database (www.proteinatlas.org).

### 2.3 Functional enrichment analysis

A list of the most relevant genes or cell clusters characteristic of STRN3 was uploaded to the Database for Annotation, Visualization and Integrated Discovery (DAVID, v2022q4). Gene Ontology (GO) analysis and Kyoto Encyclopedia of Genes and Genomes (KEGG) pathway analysis enrichment results were then downloaded, and top 7 genes in ascending order of *p*‐value (*p* < 0.05) were presented in this research.

### 2.4 Gene set variation analysis (GSVA)

The gene list of relevant bio‐function processes and signal pathway based on the GO analysis was downloaded from the AmiGO 2 portal (https://amigo.geneontology.org/amigo). The functional enrichment score of each HCC sample from the TCGA database was calculated using the R packages (GSVA 1.47.0). The heatmap of enrichment results was drawn through the R package (pheatmap 1.0.12). The correlation between STRN3 and relevant bio‐function processes and signal pathway was determined by Pearson correlation analysis.

### 2.5 Single‐cell sequencing analysis

R package (Seurat 4.3.0, dplyr 1.1.0, patchwork 1.1.2) was used to perform single‐cell sequencing data analysis, including data normalization, dimensionality reduction, PCA analysis, Umap/Tsne clustering and differential expression.

### 2.6 Cell lines and cell transfection

Human hepatoma cell lines, LM3, MHCC 97H and MHCC 97 L were obtained from the Liver Cancer Institute & Zhong Shan Hospital of Fudan University. PLC, Huh7 and Hep3B were purchased from the Cell Bank at the Institute of Biochemistry and Cell Biology, China Academy of Science (Shanghai, China) (https://www.cellbank.org.cn). Hep3B was maintained in MEM medium (Gibco, Shanghai, China), while LM3, MHCC 97H, MHCC 97 L, PLC and Huh7 were maintained in DMEM (Gibco, Shanghai, China) supplemented with 10% fetal bovine serum (FBS), 100 U/mL of penicillin sodium and 0.1 mg/mL of streptomycin sulfate at 37°C in humidified air containing 5% carbon dioxide.

The small/short interfering RNA (siRNA) designed for STRN3 interference and plasmids was purchased from GenePharma, Shanghai, China. The sequences of STRN3 siRNAs were: (1) 5′‐GGACUUAGUAAGAAGAAUATT‐3′; (2) 5′‐GCACUUACAAUGGAGAUAATT‐3′; (3) 5′‐GCAGACUUGACGGUAACAATT‐3′, and the negative control (Neg Con) was: 5′‐UUCUCCGAACGUGUCACGUTT‐3′. The siRNAs and vectors were both transfected into cells using jetPRIME (Polyplus, France) according to the manufacturer's instructions.

### 2.7 RNA isolation and real‐time quantitative reverse transcription‐polymerase chain reaction (qRT‐PCR) assay

Total RNA was isolated using RNA Extraction Kit (Promega, China) according to the manufacturer's instructions. Then, the isolated RNA was reverse transcribed into cDNA using Reverse Transcription Mix (Promega, China). Quantitative real‐time PCR was performed with Gentier 96E PCR machine (Tianlong, China). The primer sequences of STRN3 were: Forward primer (AGTGGGCTCGGTTCGAGAT) and Reverse primer (CTTGACCTTTTCTTTCGCCTTG).

### 2.8 Western Blotting

All cells were lysed with RIPA Lysis Buffer (Beyotime, China) containing 1% PMSF. Proteins were then separated using SDS‐PAGE gel and transferred onto PVDF membrane (Millipore Corp, USA). The membrane was blocked with 5% non‐fat dry milk for 1 h and further incubated overnight at 4°C with primary antibodies against STRN3 (Invitrogen, USA), YAP, p‐YAP(Ser127), MST1, p‐MST1/2(Thr183/Thr180) and GAPDH (CST, USA). The very next day, the membranes were washed three times with TBST and incubated with a horseradish peroxidase‐conjugated secondary antibody for 2 h, respectively. All protein bands were visualized by the ECL method.

### 2.9 Immunofluorescence (IF)

The cells were fixed using 4% paraformaldehyde for at least 30 min and Triton was used for 20 min to permeabilize the cell membranes. After fixation and permeabilization, the cells were incubated with primary antibodies overnight at 4°C, The very next day, cells were washed three times with PBST and incubated with fluorescently labelled secondary antibody at concentrations recommended. DAPI dyes were used for the staining of cell nuclei.

### 2.10 Cell counting kit‐8 (CCK8) assay

In this study, 1000 cells were seeded in 96‐well plates, and each sample was replicated in six wells for consistency. The plates were incubated in a cell culture incubator for a certain period of time (37°C, 5% CO_2_). Then, 10 μL of CCK‐8 solution (Beyotime, China) was added into each well, and the absorbance was measured at 450 nm with a microplate reader after 2‐h incubation.

### 2.11 Colony formation assay

Five hundred cells were seeded in six‐well plates for each experiment group. The medium was changed every 3 days and cell status was observed everyday. The assay is maintained at least for 14 days or until the majority of single clones have more than 50 cells.

### 2.12 Cell apoptosis assay

Cells from different groups were seeded into 24‐well plates and cultured for 48 h. Then, the cells were harvested, resuspended in binding buffer, and treated with the Annexin V‐FITC Apoptosis Detection Kit (BD, USA) according to the manufacturer's instructions. Briefly, 5 μL FITC‐labelled Annexin V and 5 μL PI solution were added to each sample, and the cells were incubated for 15 min at room temperature, and then analysed by flow cytometry (Canto II, BD, USA).

### 2.13 Wound healing assay

A 200 μl pipette tip perpendicular to the surface of the cells, slid from one end of the well to the other. Created a horizontal line on the back of the well as vertically as possible, keeping the pipette tip vertical and not tilting. At this time, the surface of the cell could be seen clearly on the surface of the culture dish with a ‘#’ shaped scratch. Then, take the scratched cells at 0 h, 24 h and 48 h and observe the width of the scratches in different treatment groups.

### 2.14 Invasion assay

Cell invasion ability was performed using a transwell chamber (Corning, USA). Approximately 10^5^ cells were seeded into the top chamber, while complete medium was added to the lower chamber. After certain time of incubation, cells on the upper surface were removed and the lower surface of the membrane was stained with Crystal Violet Staining Solution (Beyotime, China), photographed and counted under a microscope (×20 magnification) in 5 fields. The averages of all experiments were calculated from triplicate samples.

### 2.15 Multiple immunofluorescence analysis and immunohistochemical staining analysis

The multiple immunofluorescence (mIF) procedure was performed following the manufacturer's instructions (Servicebio, Wuhan, China). The slides were scanned and imaged using the 3D HISTECH Panoramic Scanner (Panoramic DESK, P‐MIDI, P250, Hungary), and the results were analysed by two experienced researchers for quantification of positively stained cells.

Immunohistochemical staining was performed using a specific primary antibody against STRN3, and two pathologists independently scored the STRN3 immunostaining. The immunostaining proportion and intensity were used to determine the expression of STRN3 in the tissue samples. The proportion scores were as follows: 0, <10% positive cells; 1, 10%–40% positive cells; 2, 40%–70% positive cells; 3, >70% positive cells. The intensity scores for STRN3 staining were: 0, no staining; 1, weak staining; 2, moderate staining; 3, strong staining. When the sum of proportion and intensity scores was 4 or higher, the expression status was defined as ‘high expression’.

### 2.16 Statistical analysis

SPSS 19.0 and GraphPad Prism 6 were applied in this study. Log‐rank tests were performed for survival analyses in the Kaplan–Meier graph. Differences between groups were analysed using two‐tailed unpaired Student's *t*‐test, Pearson's χ^2^ test, Mann–Whitney *U* test, two‐way ANOVA or log‐rank test, as appropriate. A two‐tailed *p* < 0.05 was considered statistically significant.

## 3 RESULTS

### 3.1 High expression of STRN3 was associated with poor prognosis of HCC patients

**FIGURE 1:**
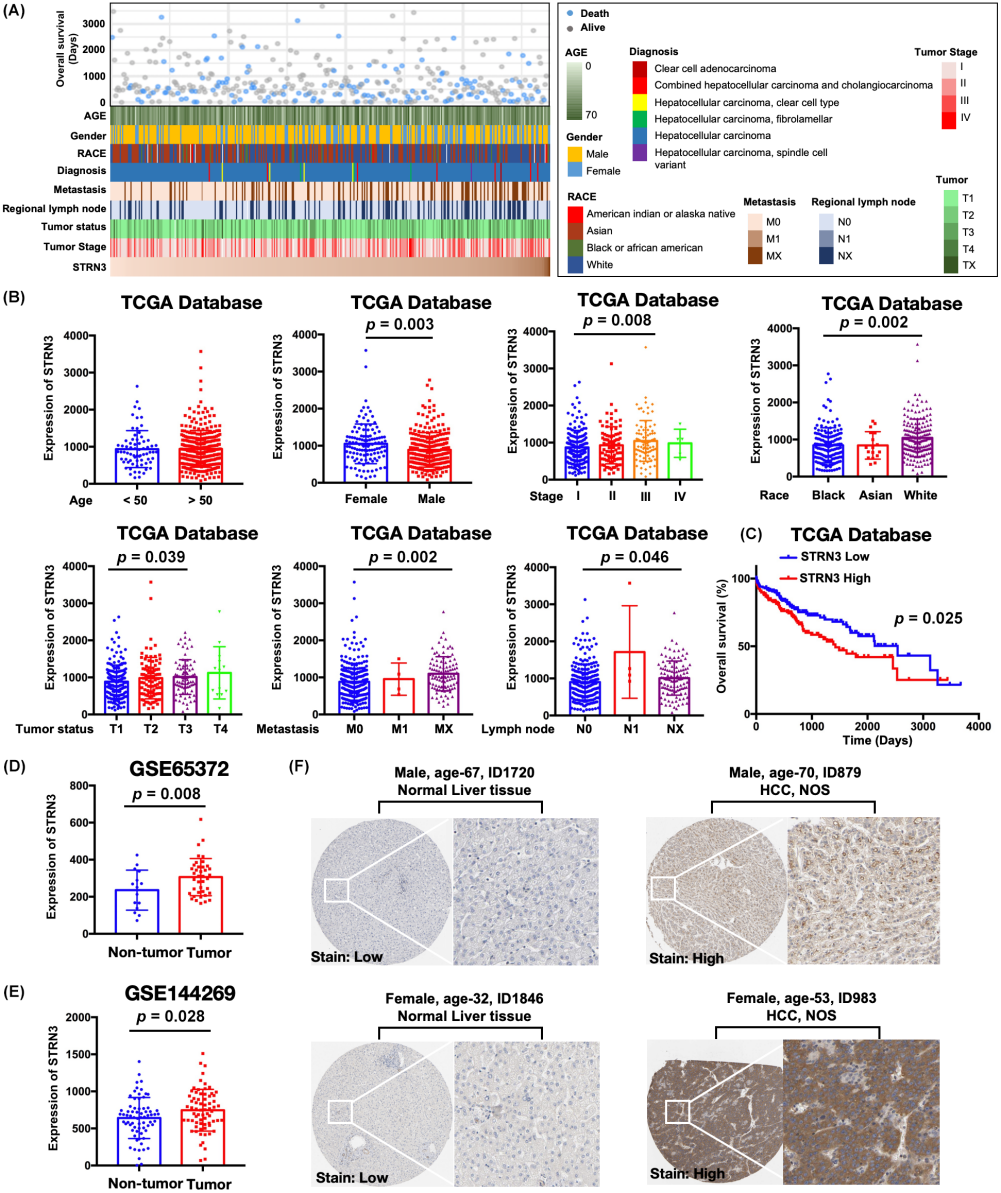
Relationship between STRN3 and clinicopathological characteristics of HCC. (A) The landscape of clinical feature of HCC patients related to STRN3 in TCGA database. (B) The expression of STRN3 in subgroup with different clinical characteristics. (C) Kaplan–Meier analysis of STRN3 for overall survival of 371 HCC patients. (D) The expression of STRN3 in 39 tumour tissues and 15 para‐tumour tissues from GSE65372. (E) The expression of STRN3 in 70 tumour tissues and 70 para‐tumour tissues from GSE144269. (F) Demonstration of STRN3 protein expression in tumour tissue versus non‐tumour tissue using immunohistochemistry and its intracellular distribution.

Three hundred and seventy‐one HCC patients with clinical and pathological characteristics were ranked in the order of STRN3 expression level. Different attribution of age, gender, race, diagnosis, TNM and Tumour stage was presented in Figure 1A. Gender had a slight effect on STRN3 expression, with females showing a higher level of STRN3 enrichment in tumour tissue than males (*p* = 0.003). Moreover, there was an association between STRN3 expression and Tumour stage, with patients at an advanced stage (Stage III) having a higher level of STRN3 expression (*p* = 0.008). As for the patients who were diagnosed as Stage IV, the number enrolled was quite small which might have had some impact on the statistics. On the other hand, we observed the expression of STRN3 in tumour tissues of White patients is higher than that of African American patients, and it also had an association with tumour status (*p* = 0.039) (Figure 1B). To further investigate the association between STRN3 expression and patient survival, we stratified the 371 HCC patients into high and low STRN3 expression groups based on the median expression level of STRN3. The Kaplan–Meier survival curve showed that patients with higher STRN3 expression had a significantly worse overall survival (OS) outcome than those with lower STRN3 expression (*p* = 0.025) (Figure 1C). To validate these findings, we also analysed RNA‐seq data from both tumour tissue and normal liver tissue. Both GEO data (GSE65372, *p* = 0.008) and (GSE144269, *p* = 0.028) confirmed that STRN3 expression was higher in HCC tissues than in peritumoral liver tissues (Figure 1D,E). Additionally, immunohistochemical staining samples from the Human Protein Atlas database demonstrated the STRN3 expression pattern in normal and cancerous tissues and showed that STRN3 mainly localized to the cell cytosol and nucleoplasm (Figure 1F).

### 3.2 STRN3 is closely involved in the transcriptional regulation process in HCC

To identify genes that are most related to STRN3 in HCC, we performed Pearson correlation analysis using data from 371 HCC patients from the TCGA database. Based on the results, we performed GO and KEGG analyses to further explore the biological functions of STRN3.

**FIGURE 2:**
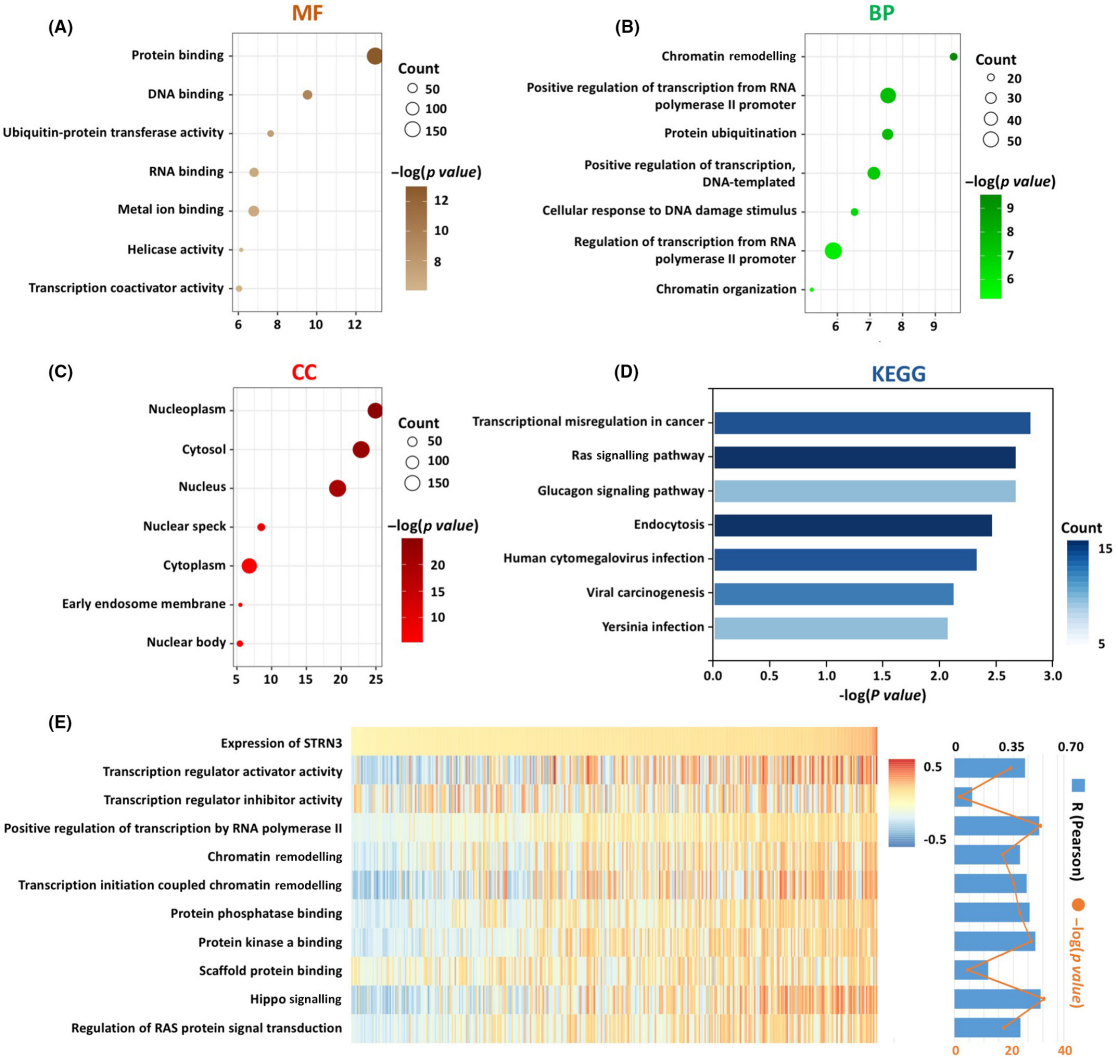
The biological process analysis of STRN3. (A–C) MF, BP and CC are mostly related to STRN3 in the TCGA database. (D) KEGG pathway analysis of STRN3 in the TCGA database. (E) Correlation analysis between STRN3 expression and related biological processes enrichment scores.

Our findings showed that the molecular function (MF) most associated with STRN3 is protein binding (Figure 2A), which is consistent with previous research. STRNs have several binding regions, including a conserved caveolin‐binding (CAV) region, a coiled‐coil domain, a calmodulin‐binding region, a CCM3‐binding region and a C‐terminal WD40‐repeat domain.^6^ These functional regions allow STRN3 to bind signalling molecules or specific proteins, making it a vital regulator of signal transduction. The biological processes (BP) analysis suggested that chromatin remodelling is most significantly related to STRN3 (Figure 2B). Chromatin remodelling means changes in the position and structure of chromatin. Slippage of nucleosomes on DNA or even removal or interchange between histones and their variants are the underlying causes. As reported, this process is mainly regulated by chromatin regulators (CRs), which are believed to have the capability to influence remarkable gene expression and inactivation changes to accelerate disease progression.^11^, ^12^ Moreover, certain proteins with specific domains, such as WD40‐repeats, can also participate in chromatin modification,^13^ which means that STRN3 might have the potential to regulate chromosomes. The GO cellular components (CC) analysis indicated that STRN3 is mainly distributed in the nucleoplasm and cytosol (Figure 2C), which is consistent with IHC results. KEGG analysis revealed that the most relevant signal pathway to STRN3 is transcriptional misregulation in cancer (Figure 2D). Previous research has shown that STRN3 acts as a regulatory subunit of PP2A, influencing the activation of the YAP‐Hippo axis.^8^, ^14^ However, it remains unknown whether STRN3 can regulate Hippo or other signalling pathways through its multiple protein binding sites in HCC, and this requires further investigation. Based on our results, we speculate that STRN3 is likely to be involved in the biological process of transcriptional regulation, and may further influence HCC development.

Next, to determine the enrichment score of important biological processes related to STRN3, we performed GSVA analysis using data from 371 HCC patients from TCGA. The results showed that STRN3 had a positive correlation with transcription regulator activator activity, transcription initiation coupled chromatin remodelling, protein phosphatase binding, protein kinase A binding, Hippo signalling and regulation of RAS protein signal transduction (Figure 2E). This suggests that in HCC, STRN3 is involved in a wide range of cell biological functions, mainly including signalling transduction, activation of signalling pathways and transcriptional regulation.

### 3.3 STRN3 knockdown inhibited cell proliferation, invasion and induced apoptosis in HCC

**FIGURE 3:**
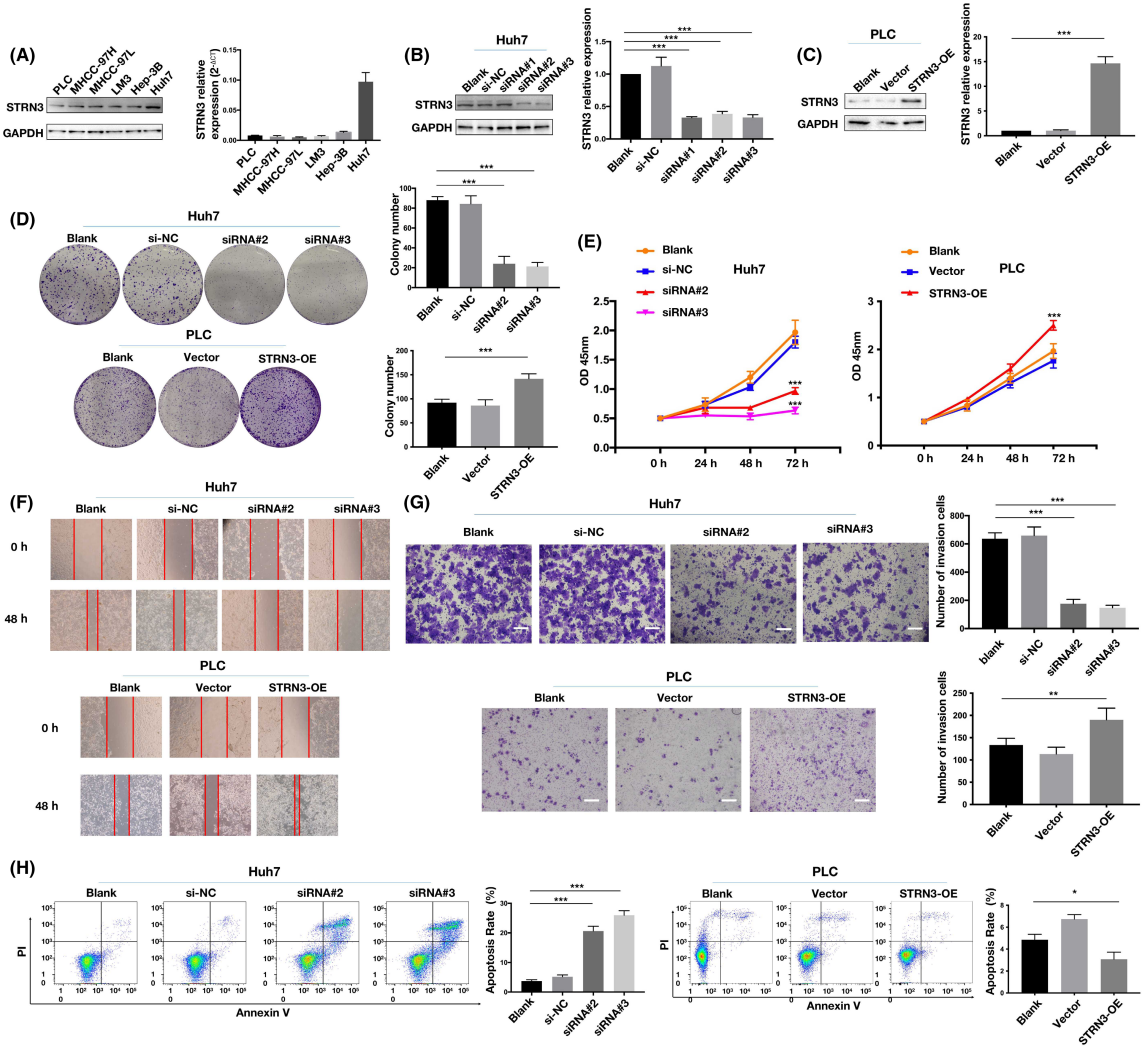
STRN3 prompted cell proliferation, invasion and anti‐apoptosis in HCC. (A) mRNA and protein expression of STRN3 in different HCC cell lines. (B–C) Efficiencies of STRN3 knockdown by siRNA and overexpression were validated by qRT‐PCR and Western blotting. (D–E) Effects of STRN3 downregulation and upregulation on cell proliferation potentials were assessed by colony‐formation assays and CCK8. (F–G) Effects of STRN3 downregulation and upregulation on cell invasion capacity were assessed by wound healing assay and invasion assay. H. Effects of STRN3 downregulation and upregulation on cell apoptosis were measured by flow cytometry. *p < 0.05; **p < 0.01; ***p < 0.001.

To investigate the biological function of STRN3 in HCC, we determined its expression levels in six HCC cell lines. Our results revealed that STRN3 was highly expressed in Huh7 cells and lowly expressed in PLC cells (Figure 3A). We then used siRNA to knock down STRN3 expression in Huh7 cells, while ectopic overexpression of STRN3 was performed in PLC. Then, we confirmed the knockdown and overexpression efficiency using qRT‐PCR and western blotting (Figure 3B,C). siRNA#2 and siRNA#3 were selected for further cell function assays. The CCK8 and colony‐formation assays demonstrated that knockdown of STRN3 significantly inhibited Huh7 cell proliferation, while after overexpressing STRN3 in PLC cells, there was a significant enhancement in their cell proliferation capability (Figure 3D,E). Furthermore, the invasion and wound healing assays showed that STRN3 is associated with cell migration capability. Knocking down STRN3 can inhibit cell migration, while after overexpressing STRN3, the invasion potential of PLC was evidently enhanced. (Figure 3F,G). The apoptosis assay also revealed a close correlation between STRN3 and cell survival status, as downregulation of STRN3 significantly promoted cell apoptosis (Figure 3H). These results collectively suggest that STRN3 plays a significant role in HCC cell proliferation and anti‐apoptosis, which contributes to tumour progression in a protective way. This is consistent with our previous finding from TCGA database analysis, which showed that high expression of STRN3 in tumour tissues was associated with poor prognosis in HCC patients.

### 3.4 STRN3 is an important upstream regulatory molecule in the Hippo signalling pathway in HCC

**FIGURE 4:**
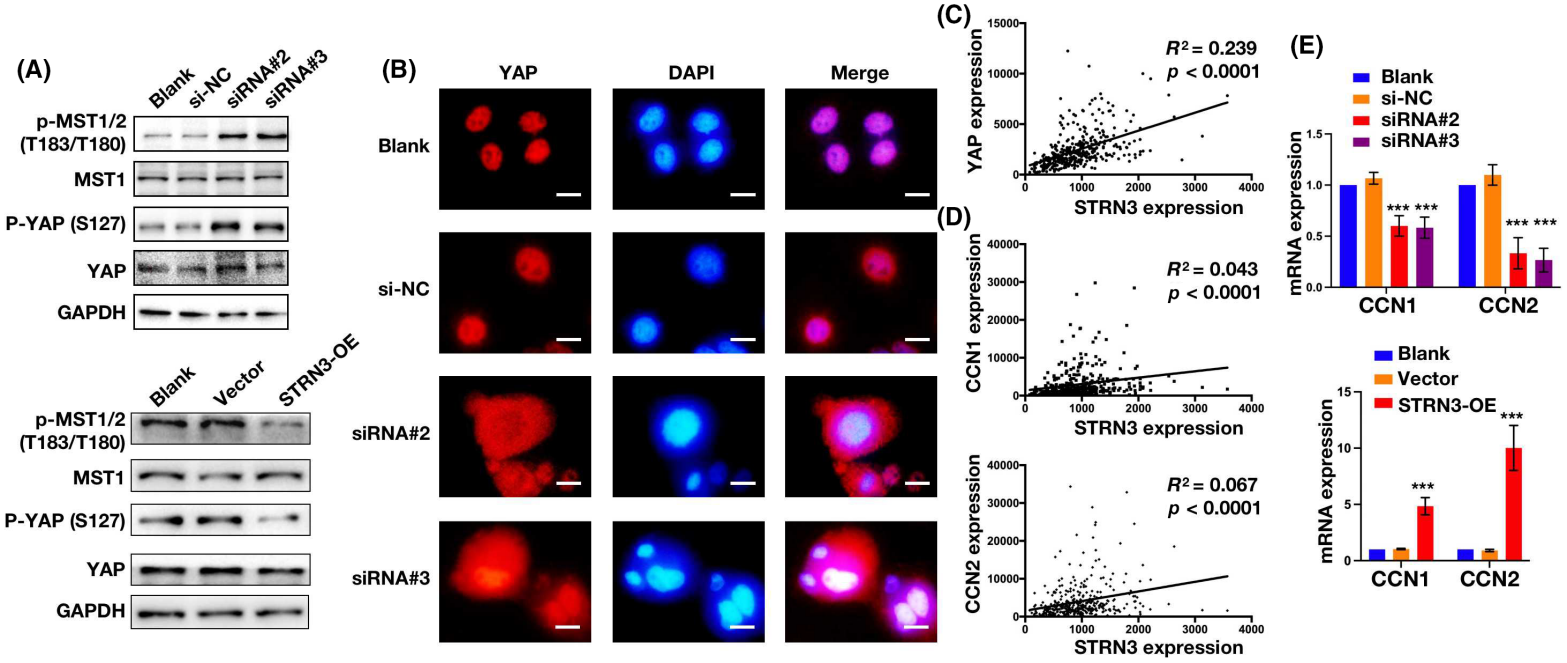
STRN3 prompted the transcriptional activity of YAP. (A) The expression of key proteins of the Hippo pathway was detected by western blotting after the knockdown and overexpression of STRN3. (B) Representative immunofluorescence staining images of YAP distribution in HCC cells after knockdown of STRN3. Scale bar: 50 μm. (C) The correlation between STRN3 and YAP with TCGA DATA. (D) The correlation between STRN3 and YAP targeted gene (CCN1 and CCN2). (E) The relative mRNA expression of CCN1 and CCN2 after knockdown and overexpression of STRN3 in HCC cell lines.

The expression and intracellular distribution of the YAP protein play a crucial role in the activation of the Hippo signalling pathway. Subsequently, we analysed the key proteins in the Hippo pathway, including MST1/2, YAP, and their respective phosphorylation levels with western blot. The results showed that the knockdown of STRN3 protein expression did not significantly affect the expression levels of MST1 and YAP in Huh7 cells. However, it significantly increased the phosphorylation levels of YAP and MST1/2, indicating that STRN3 is an important upstream regulatory molecule in the Hippo signalling pathway. Downregulation of STRN3 expression can promote Hippo pathway activation, while after overexpressing STRN3, the phosphorylation levels of YAP and MST1/2 decreased, indicating the inhibition of the Hippo pathway. (Figure 4A). Furthermore, we conducted cell immunofluorescence experiments and found that in the control group, the YAP protein was primarily localized in the cell nucleus. However, after knocking down the STRN3 protein, the YAP protein was dispersed in the cytoplasm (Figure 4B). This change in YAP protein localization further confirmed that STRN3 can regulate the intracellular localization of YAP and thus control the activation status of the Hippo pathway. We next conducted a correlation analysis of STRN3, YAP and YAP target genes CCN1 and CCN2 using the TCGA database. The results showed a significant correlation between STRN3 and YAP (*R* ^2^ = 0.239, *p* < 0.0001), as well as a clear positive correlation between STRN3 and YAP target genes (CCN1: *R* ^2^ = 0.043, *p* < 0.0001; CCN2: *R* ^2^ = 0.067, *p* < 0.0001) (Figure 4C,D). Furthermore, we conducted assessments of CCN1 and CCN2 expression in cells with STRN3 knockout or overexpression. A significant decrease was observed in the expression of CCN1 and CCN2 upon STRN3 knockout, whereas an evident increase in the mRNA levels of CCN1 and CCN2 was observed upon STRN3 overexpression (Figure 4E). Taken together, based on the aforementioned findings, we conclude that STRN3 can effectively modulate YAP's transcriptional activity. These findings provide further evidence for the importance of STRN3 in regulating the Hippo pathway.

### 3.5 The expression distribution and abundance of STRN3 and YAP in HCC cells

**FIGURE 5:**
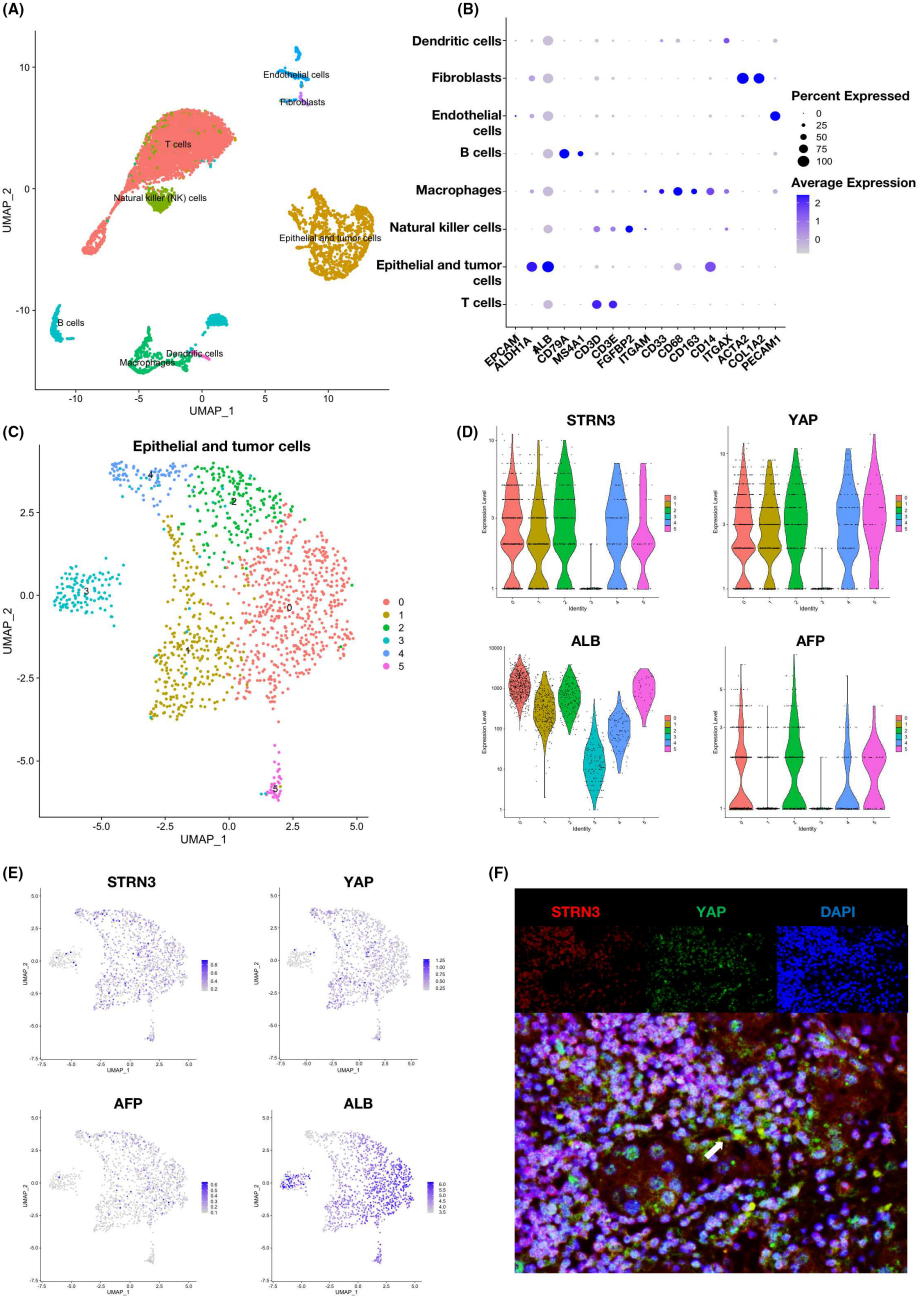
The expression pattern of STRN3 in HCC. (A) Different clusters' identification of single cells in human HCC tumour showed with UMAP plot. (B) specific cell marker for different cell types in human HCC tissues shown with a dot plot. (C) Subtypes of HCC cells from the epithelial and tumour cells cluster. (D–E) The abundance and distribution of STRN3, YAP, ALB and AFP are shown with UMAP and violin plot. (F) Representative images of multiplex immunofluorescence results in HCC patients.

To comprehensively characterize the correlation between STRN3 and YAP in HCC, we analysed the single‐cell RNA (scRNA) sequencing data from NCBI Geo DataSets. Principal component analysis and a uniform manifold approximation and projection (UMAP) were used for clustering and data dimensionality reduction. All cells were eventually assigned to 8 distinct cell types (Figure 5A) with specific markers: EPCAM, ALDH1A1 and ALB for epithelial and tumour cells; CD79A and MS4A1 for B cells; CD3D and CD3E for T cells; FGFBP2 for natural killer (NK) cells; ITGAM, CD33, CD68, CD14 and CD163 for monocytes and macrophages; ITGAX for dentritic cells; ACTA2 and COL1A2 for fibroblasts; PECAM1 for endothelial cells (Figure 5B). The expression of specific markers mentioned above in every cell cluster was shown in Figures S1, S2. Next, we isolated the epithelial and tumour cells cluster for further analysis (Figure 5C). ALB was used to define liver cells, while AFP was used to define HCC cells. By examining the distribution of STRN3 expression across different clusters, we found that STRN3 is enriched in clusters 0, 1, 2, 4 and 5, with similar abundance and distribution as YAP. Furthermore, we observed a significant overlap between the distribution ranges of STRN3 and AFP (Figure 5D,E). To further validate the results obtained from bioinformatics analysis, we performed a multi‐immunofluorescence assay with tumour tissues from HCC patients. The co‐expression of STRN3 and YAP in cells can be clearly observed, and the result indicated the expression of the two proteins had a significant correlation(Figure 5F). These results suggested a close correlation between the expression of STRN3 and YAP at the cellular level and a significant increase in STRN3 expression in HCC cells.

### 3.6 STRN3 is upregulated in liver cancer tissues and associated with poor prognosis

**FIGURE 6:**
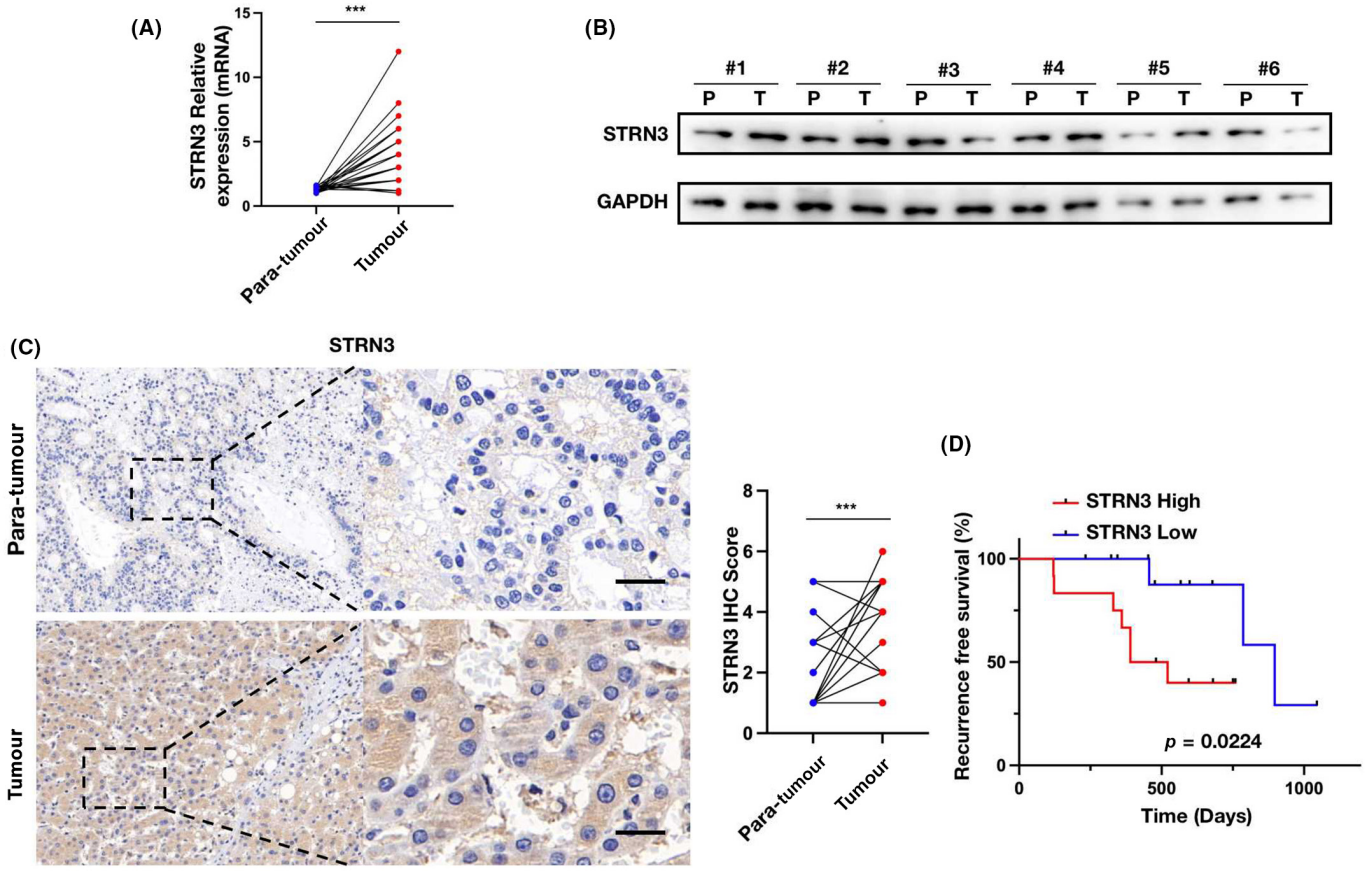
Upregulation of STRN3 in HCC tissues. (A) RT‐PCR assay of STRN3 mRNA expression in 24 HCC tissues and matched non‐tumour liver specimens. (B) The protein expression of STRN3 in 6 pairs of HCC tumour tissues (T) and corresponding non‐tumour liver tissues (P). (C) Representative immunohistochemistry staining on STRN3 in 24 HCC patients. (D) Kaplan–Meier analysis of the recurrence‐free survival of HCC patients in STRN3 low group and STRN3 high group; Scale bar: 100 μm.

We collected a small cohort of HCC patients and conducted tests on the mRNA and protein expression of STRN3 in tumour and adjacent non‐cancerous tissues. Through experiments, we found that the mRNA expression of STRN3 in the adjacent tissues of these 24 liver cancer patients was significantly lower than its expression in the tumours (Figure 6A). Additionally, Western blot was used to examine the expression of STRN3 protein in 6 patients' cancer and adjacent non‐cancerous tissues. The results visually demonstrated that the expression of STRN3 in the adjacent tissues was significantly weaker than in the tumours (Figure 6B).

Furthermore, immunohistochemistry results from the 24 patients also indicated a significant difference in STRN3 expression between the adjacent non‐cancerous tissues and the tumours (Figure 6C). Finally, we evaluated the prognosis of the 24 patients using Kaplan–Meier analysis, which suggested that patients with high STRN3 expression in tumour tissues were more likely to experience recurrence after undergoing surgical treatment (Figure 6D).

## 4 DISCUSSION

HCC is a highly prevalent malignancy worldwide, characterized by high mortality and recurrence rates. Traditional treatment options mainly involve surgical resection, radiation therapy, chemotherapy and transarterial chemoembolization (TACE).^15^ However, the therapeutic effects are limited. In recent years, breakthroughs in gene‐related research and technology have led to the emergence of targeted drugs for HCC, such as multi‐kinase inhibitors sorafenib and tyrosine kinase receptor inhibitors lenvatinib.^16^ These drugs have effectively prolonged the survival time of HCC patients and improved their prognosis. However, there is still a significant proportion of patients who exhibit poor response to these drugs or develop resistance after initial treatment.^17^, ^18^ Therefore, the discovery of new and applicable drug targets remains a critical component of clinical HCC management.

The Hippo signalling pathway is an important tumour suppressor that is evolutionarily conserved. It plays a critical role in restricting cell proliferation and survival in embryonic development, and once it is disrupted, it can lead to tumorigenesis.^19^ The key components of the Hippo signalling pathway include serine/threonine protein kinases 4/3 (MST1/2), large tumour suppressor kinases (LATS) 1/2, the adaptor protein Mps one binder 1 (Mob1), transcriptional coactivator yes‐associated protein (YAP) and WW domain‐containing transcription regulator protein 1 (WWTR1 or commonly known as TAZ).^20^ Previous research reported YAP, as a vital transcription coactivator, was hyper‐activated in human malignancies,^21^ considered to play a crucial role in tumorigenesis, as its activation is believed to enhance tumour proliferation, migration and promote drug resistance. High expression of YAP in tumour tissue is often associated with poor prognosis in patients.^22^, ^23^ In this study, we found an important regulatory protein in upstream of the Hippo pathway named STRN3. Analysis of TCGA and GEO databases, along with the immunohistochemical staining analysis revealed that compared to normal liver tissue, STRN3 was significantly upregulated in tumour tissue. Furthermore, follow‐up information of 371 HCC patients from the TCGA database and 24 HCC patients enrolled in the research were analysed and found that patients with high STRN3 expression had poorer outcomes, confirming its' clinical value in predicting the prognosis of HCC patients. Additionally, we used bioinformatics to analyse the biological function of STRN3 protein and found that it is mainly associated with transcriptional abnormalities in tumours, and the results also indicated that there was a high correlation between STRN3 and the Hippo pathway in HCC. Subsequently, cell experiments further confirmed that STRN3 could promote the dephosphorylation of MST1/2 protein and YAP protein to inhibit the Hippo pathway, regulate the nuclear entry of YAP protein, and promote the transcription of related genes. As reported, STRN3 is a regulatory subunit of PP2A and forms the PP2A holoenzyme by binding to two additional subunits of PP2A (PP2Aa and PP2Ac) through its coiled‐coil domain, and further forming the STRIPAK complex.^24^ Several research had already highlighted the correlation between STRIPAK complex and the Hippo pathway in tumorigenesis. Chen revealed active RhoA bound and dissociated rhophilin and NF2/Kibra from STRIPAK, inducing the STRIPAK phosphatase to catalyse the dephosphorylation of MST1/2 and MAP4Ks by binding to the PP2AC subunit. While Kim found that expression of small T antigen (ST) induced increased interaction between MAP4K4 and the STRIPAK complex, which in turn reduced the phosphorylation and activity levels of MAP4K4, leading to increased activity of YAP1.^25^, ^26^ Our research further elucidated the important role of STRN3, as a great component of STRIPAK itself in the dephosphorylation of MST1/2 and YAP in HCC, as well as its biological impact on tumour cell proliferation and migration. This provides a theoretical basis for STRN3 as a therapeutic target in the future.

However, there are still several limitations in our study. First, we have not elucidated the specific mechanisms by which STRN3 affects the changes in MST1/2 and YAP phosphorylation, and whether STRN3 knockdown would influence to the structure and function of PP2A and the STRIPAK complex. Second, we have not conducted animal experiments to demonstrate the effect of STRN3 on tumour development in vivo. Third, the number of HCC patients enrolled in the research was insufficient, and their prognosis survival time was not long enough. In future studies of STRN3, we will address these issues and further investigate the biological role of STRN3 in HCC in more detail to improve the precision of this study.

## AUTHOR CONTRIBUTIONS

**Jie Zhu:** Data curation (equal); funding acquisition (equal); investigation (equal); software (equal); writing – original draft (equal). **Wenjia Tang:** Investigation (equal); methodology (equal); writing – original draft (equal). **Peiqi Fang:** Investigation (equal); methodology (equal). **Chong Wang:** Data curation (equal); validation (equal). **Meixiu Gu:** Data curation (equal); validation (equal). **Wenjing Yang:** Data curation (equal); supervision (equal); validation (equal). **Baishen Pan:** Conceptualization (equal); supervision (equal). **beili wang:** Conceptualization (equal); funding acquisition (equal); project administration (equal); supervision (equal); validation (equal). **Wei Guo:** Conceptualization (equal); funding acquisition (equal); project administration (equal); supervision (equal).

## FUNDING INFORMATION

This research was funded by the National Natural Science Foundation of China (82202608, 82172348, 81902139, 81972000 and 82102483), the Constructing Project of Clinical Key Disciplines in Shanghai (shslczdzk03302), the Specialized Fund for the Clinical Researches of Zhongshan Hospital, Fudan University (2020ZSLC54), the Key Medical and Health Projects of Xiamen (YDZX20193502000002) and Shanghai Baoshan Medical Key Specialty(BSZK2023A18).

## CONFLICT OF INTEREST STATEMENT

The authors have no conflict of interest.

## ETHICS STATEMENT

Approval of the research protocol by an Institutional Reviewer Board: This study was approved by the Ethics Committee of Zhongshan Hospital, Fudan university.

## INFORMED CONSENT

All HCC samples were collected on condition of informed consent.

## Supporting information


**Figure S1.** UMAP plots showed specific marker genes in different cell clusters.

**Figure S2.** Violin plot showed specific marker genes in different cell clusters.

## Data Availability

The data and materials used in this study can be obtained upon request from the authors. To request access to the data and materials, please contact the corresponding author. Once the request is reviewed and approved, the data and materials will be made available to you.
