# Supplementary figures and images for: STRN3 promotes tumour growth in hepatocellular carcinoma by inhibiting the hippo pathway
Source: J Cell Mol Med. 2024 Mar 1;28(6):e18147. doi: 10.1111/jcmm.18147 (PMC10907822; doi:10.1111/jcmm.18147)

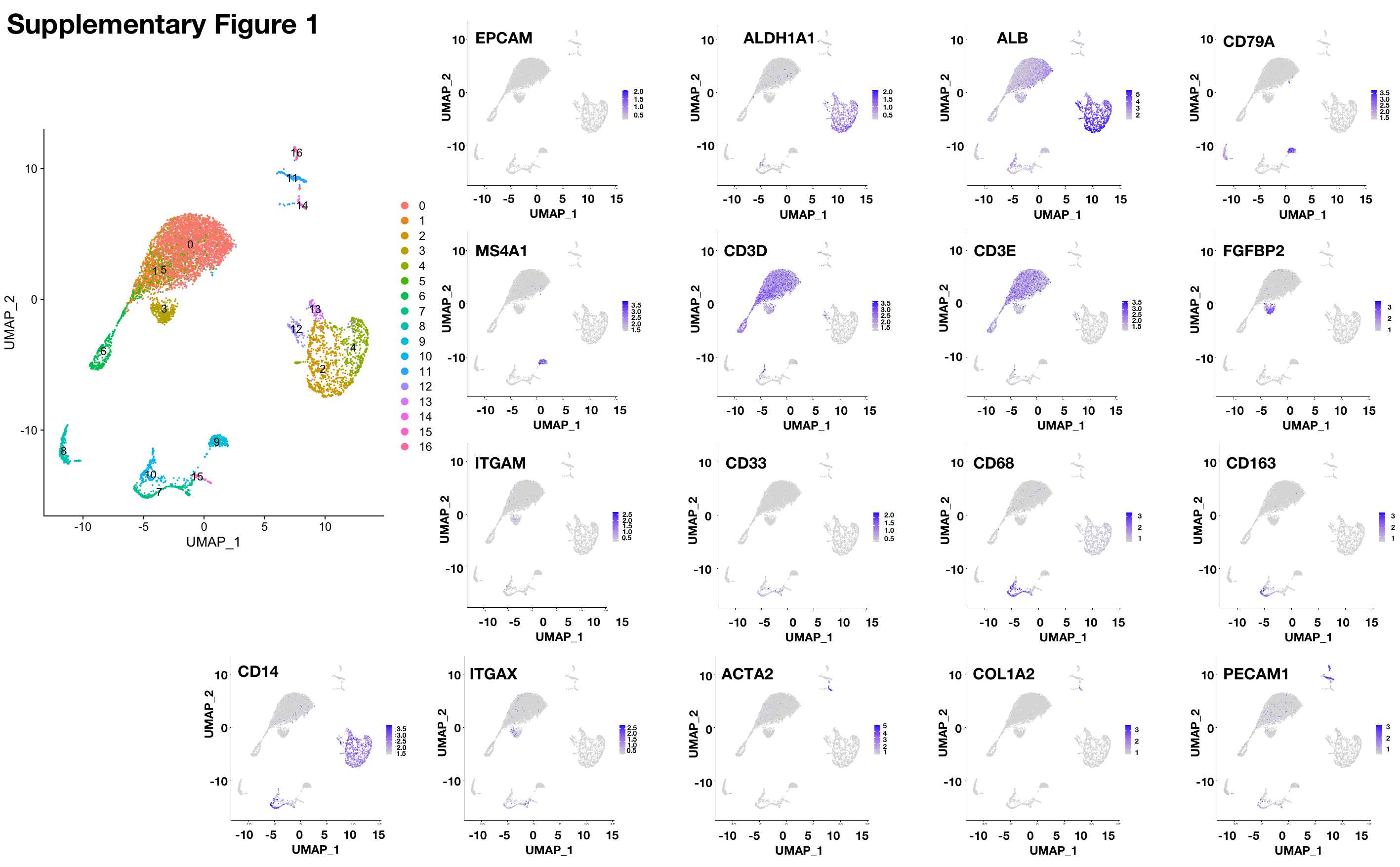

Supplement: Supplementary file 1 — Figure S1. UMAP plots showed specific marker genes in different cell clusters. [file JCMM-28-e18147-s002.jpg]

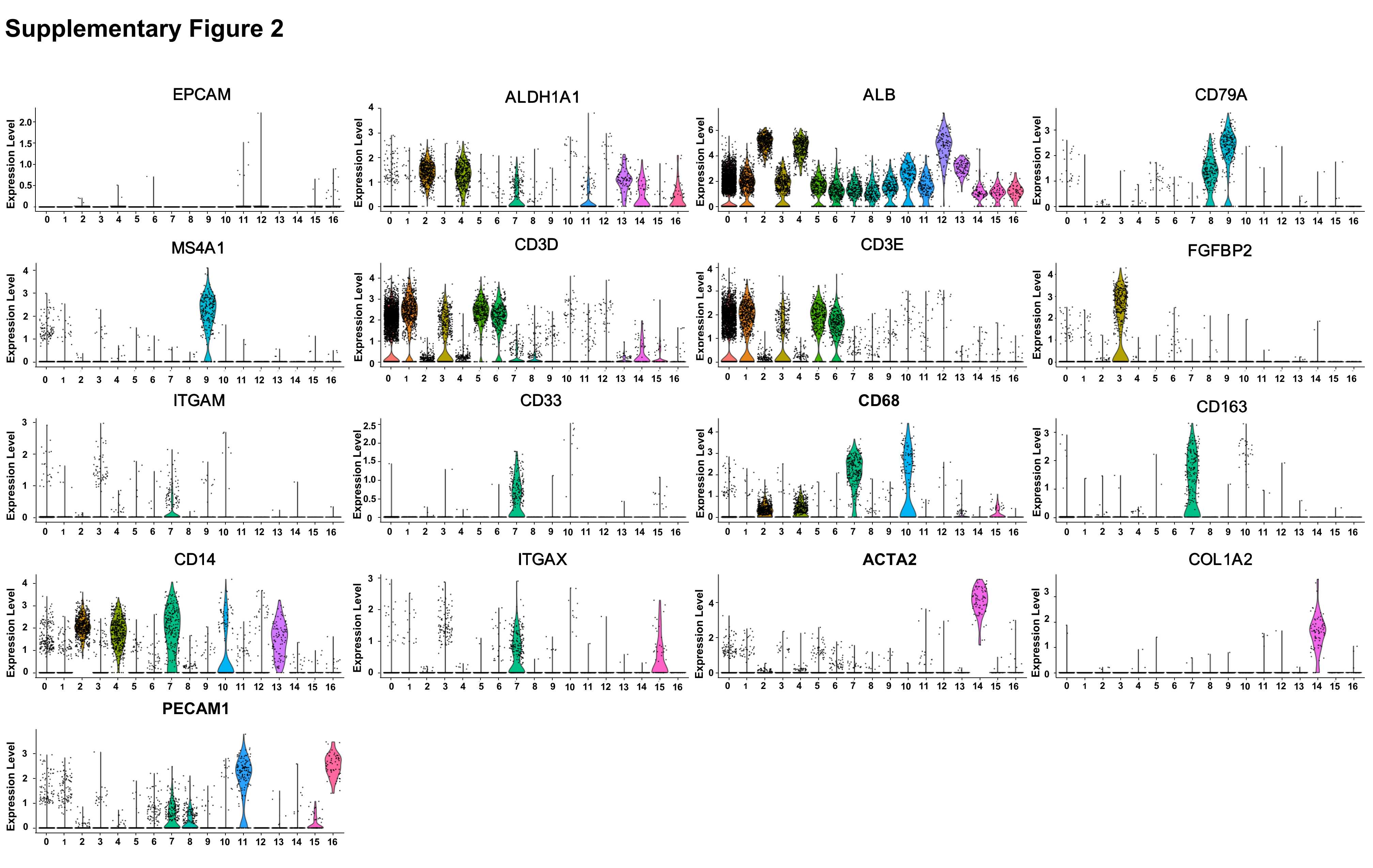

Supplement: Supplementary file 2 — Figure S2. Violin plot showed specific marker genes in different cell clusters. [file JCMM-28-e18147-s001.jpg]
